# Supplementary figures and images for: A comparative analysis of the complete chloroplast genome sequences of four peanut botanical varieties
Source: PeerJ. 2018 Jul 31;6:e5349. doi: 10.7717/peerj.5349 (PMC6074784; doi:10.7717/peerj.5349)

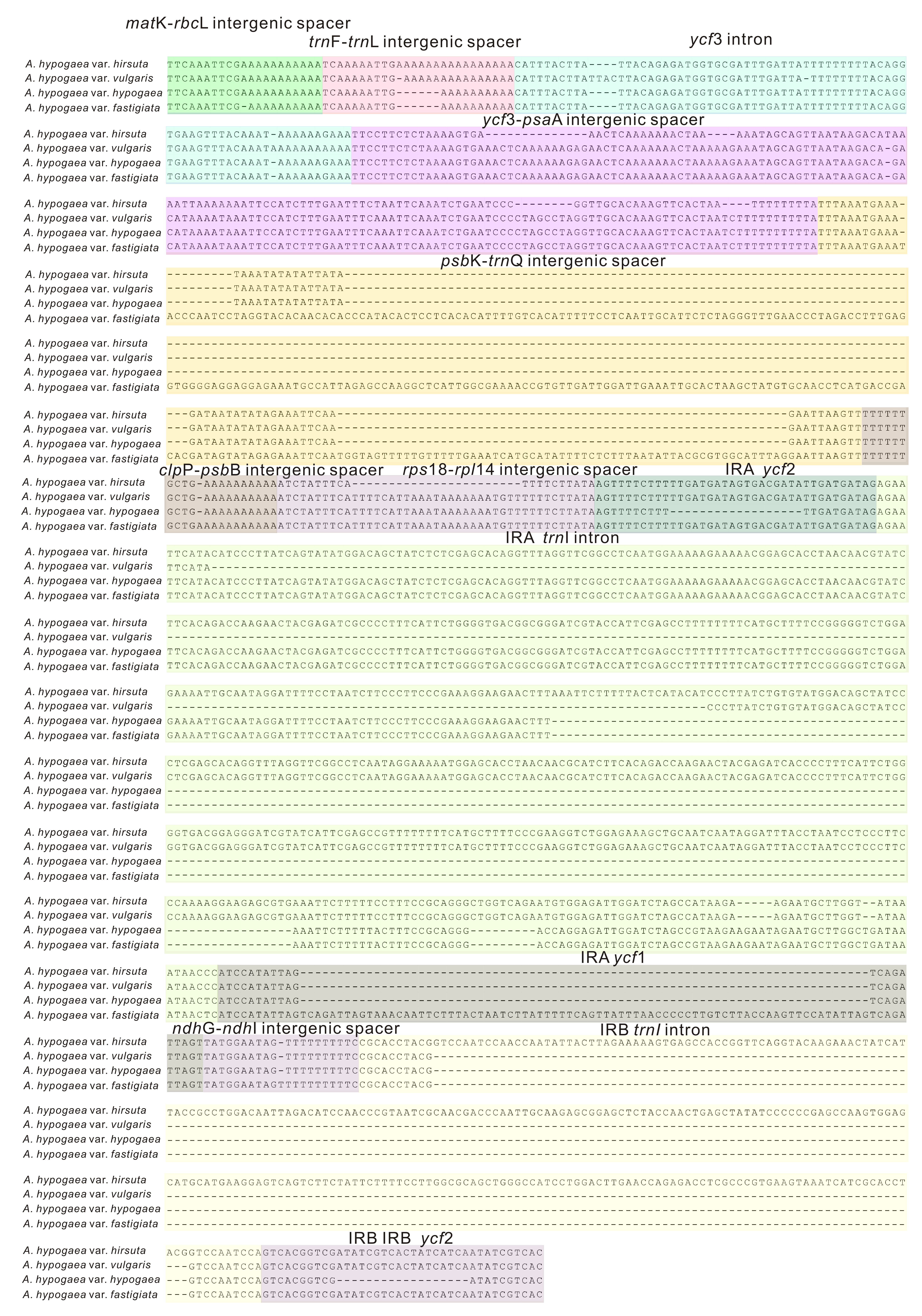

Supplement: Supplemental Information 1 [file peerj-06-5349-s001.png]
