# Supplementary material for: A comparative analysis of the complete chloroplast genome sequences of four peanut botanical varieties
Source: PeerJ. 2018 Jul 31;6:e5349. doi: 10.7717/peerj.5349 (PMC6074784; doi:10.7717/peerj.5349)
Supplement: Supplemental Information 2 [file peerj-06-5349-s002.docx]

**Table S1** SNP distribution of peanut cp genome.

| **NO.** | ***fastigiata*** | | ***hirsuta*** | | ***hypogaea*** | | ***vulgari*** | | **Annotation** |
| --- | --- | --- | --- | --- | --- | --- | --- | --- | --- |
|  | **SNP** | **Position** | **SNP** | **Position** | **SNP** | **Position** | **SNP** | **Position** |  |
| **1** | A | 5069 | C | 5070 | A | 5070 | C | 5070 | *trnK*-UUU *rbcL* spacer |
| **2** | T | 14851 | C | 14858 | C | 14852 | C | 14857 | *trnL*-UAA *trnT*-UGU spacer |
| **3** | C | 16886 | C | 16893 | C | 16887 | A | 16895 | *ycf3* intron |
| **4** | G | 16930 | G | 16937 | G | 16931 | A | 16940 | *ycf3* intron |
| **5** | G | 19218 | A | 19208 | G | 19219 | G | 19228 | *ycf3 psaA* spacer |
| **6** | C | 19220 | A | 19210 | C | 19221 | C | 19230 | *ycf3 psaA* spacer |
| **7** | A | 19223 | T | 19213 | A | 19224 | A | 19233 | *ycf3 psaA* spacer |
| **8** | T | 19227 | A | 19217 | T | 19228 | T | 19237 | *ycf3 psaA* spacer |
| **9** | A | 19248 | T | 19238 | A | 19249 | A | 19258 | *ycf3 psaA* spacer |
| **10** | A | 19300 | C | 19282 | A | 19301 | A | 19310 | *ycf3 psaA* spacer |
| **11** | G | 19301 | A | 19283 | G | 19302 | G | 19311 | *ycf3 psaA* spacer |
| **12** | G | 19434 | T | 19416 | G | 19435 | G | 19444 | *ycf3 psaA* spacer |
| **13** | C | 19480 | A | 19462 | C | 19481 | C | 19490 | *ycf3 psaA* spacer |
| **14** | T | 19834 | A | 19812 | T | 19835 | T | 19844 | *psaA* |
| **15** | G | 56536 | A | 56325 | A | 56348 | A | 56357 | *psbK trnQ*-UUG spacer |
| **16** | G | 56541 | T | 56330 | T | 56353 | T | 56362 | *psbK trnQ*-UUG spacer |
| **17** | C | 57530 | G | 57319 | C | 57342 | G | 57351 | *trnQ*-UUG *accD* spacer |
| **18** | C | 62221 | C | 62010 | T | 62033 | C | 62042 | *cemA petA* spacer |
| **19** | A | 104980 | A | 104684 | A | 104712 | T | 104736 | *trnI*-GAU intron |
| **20** | A | 104983 | A | 104687 | A | 104715 | G | 104739 | *trnI* -GAU intron |
| **21** | T | 104998 | T | 104702 | T | 104730 | C | 104754 | *trnI* -GAU intron |
| **22** | G | 105029 | G | 104733 | G | 104761 | A | 104785 | *trnI* -GAU intron |
| **23** | C | 105042 | C | 104746 | C | 104774 | T | 104793 | *trnI* -GAU intron |
| **24** | T | 105059 | T | 104763 | T | 104791 | C | 104810 | *trnI* -GAU intron |
| **25** | G | 105088 | G | 104792 | G | 104820 | A | 104839 | *trnI* GAU intron |
| **26** | C | 105112 | C | 104816 | C | 104844 | A | 104863 | *trnI* -GAU intron |
| **27** | G | 105153 | A | 105118 | G | 104885 | A | 104906 | *trnI* -GAU intron |
| **28** | G | 105154 | A | 105119 | G | 104886 | A | 104907 | *trnI* GAU intron |
| **29** | A | 105167 | G | 105132 | A | 104899 | G | 104920 | *trnI* -GAU intron |
| **30** | T | 105168 | C | 105133 | T | 104900 | C | 104921 | *trnI* -GAU intron |
| **31** | T | 105182 | A | 105151 | T | 104914 | A | 104939 | *trnI* -GAU intron |
| **32** | T | 105190 | C | 105159 | T | 104922 | C | 104947 | *trnI* -GAU intron |
| **33** | G | 105200 | A | 105169 | G | 104932 | A | 104957 | *trnI* -GAU intron |
| **34** | A | 105210 | G | 105179 | A | 104942 | G | 104967 | *trnI* -GAU intron |
| **35** | T | 105212 | G | 105181 | T | 104944 | G | 104969 | *trnI* -GAU intron |
| **36** | T | 105214 | G | 105183 | T | 104946 | G | 104971 | *trnI* -GAU intron |
| **37** | A | 105226 | C | 105195 | A | 104958 | C | 104983 | *trnI* -GAU intron |
| **38** | C | 105240 | T | 105218 | C | 104972 | T | 105006 | *trnI* -GAU intron |
| **39** | C | 105241 | G | 105219 | C | 104973 | G | 105007 | *trnI* -GAU intron |
| **40** | A | 105242 | T | 105220 | A | 104974 | T | 105008 | *trnI* -GAU intron |
| **41** | G | 105260 | A | 105238 | G | 104992 | A | 105026 | *trnI* -GAU intron |
| **42** | A | 105269 | G | 105242 | A | 105001 | G | 105030 | *trnI* -GAU intron |
| **43** | T | 105270 | A | 105243 | T | 105002 | A | 105031 | *trnI* -GAU intron |
| **44** | C | 105282 | T | 105255 | C | 105014 | T | 105043 | *trnI* -GAU intron |
| **45** | T | 105294 | C | 105265 | T | 105026 | C | 105053 | *trnI* -GAU intron |
| **46** | G | 115635 | T | 115529 | T | 115290 | T | 115317 | *rpl32* *trnL*-UAG spacer |
